# Crude and Adjusted Prevalence of Sleep Complaints in Mexico City

**DOI:** 10.5935/1984-0063.20170020

**Published:** 2017

**Authors:** Alejandro Jiménez-Genchi, Jorge Caraveo-Anduaga

**Affiliations:** 1 Servicios Clínicos, Instituto Nacional de Psiquiatría Ramón de la Fuente; 2 División de Investigaciones Epidemiológicas y Sociales, Instituto Nacional de Psiquiatría Ramón de la Fuente

**Keywords:** Prevalence, Epidemiology, Sleep disorders, Mexico

## Abstract

**OBJECTIVE:**

To estimate the crude prevalence rates of several sleep complaints and the prevalence for each one adjusted for the coexistence of symptoms in other sleep domains in a representative sample of adult individuals from Mexico City.

**METHODS:**

A probabilistic sample of 1933 adult individuals living in Mexico City was surveyed using fourteen questions of the Sleep Disorders Questionnaire to assess sleep-related symptoms and sleep complaints. Estimates of crude prevalence rates for each sleep disturbance and adjusted for a score ≥ the 80th. percentile in the questionnaire were calculated.

**RESULTS:**

The following prevalence rates were found: insomnia 39.7%; excessive diurnal sleepiness (EDS) 20.9%; obstructive sleep apnea syndrome (EDS plus snoring) 7.7%; habitual snoring 9.9%; restless legs syndrome (RLS) 4.4%; narcolepsy 0.9%; sleep paralysis (SP) 13.2%; and hypnotic use 1.2%. When prevalence rates were calculated accounting for symptoms in other sleep domains, notable reductions were observed in complaints of insomnia (17.3%), EDS (10.3%), and SP (8.7%), while minor decreases were observed for complaints of snoring (7.4%), OSAS (5%), and RLS (3.8%); narcolepsy prevalence practically did not change (0.9%).

**CONCLUSIONS:**

Sleep complaints are highly prevalent in Mexican adult population. More than a half of the individuals with a given sleep disturbance have a global sleep deterioration associated to psychosocial and health impairments.

## INTRODUCTION

The high frequency of sleep problems in general population means that a significant amount of people are exposed to the various associated consequences, such as an increased risk of traffic-^1,2^ and job-related accidents^3^, an increased risk of psychiatric^4^ and non-psychiatric morbidity^5-7^, a decreased productivity^3,8^, and a decreased quality of life^8-11^.

The epidemiologic approach to the study of sleep disorders rarely considers the high comorbidity between sleep disorders or between disturbances in different sleep domains (e.g. sleep apnea with insomnia symptoms); this is particularly relevant because the adverse impact on health and quality of life might be greater. Thus, while knowledge about the distribution of sleep disorders among the general population is critical for the development and implementation of health policies, a closer look at the magnitude of the problem may be obtained through the estimate of population rates with a given sleep disorder taking into account the coexistence of other sleep disturbances and the association with adverse psychosocial and health outcomes. This approach to the study of sleep disorders epidemiology has not been previously performed in Mexico, where two population-based studies have been conducted so far^12,13^; however, they included non-representative samples of adult population and some sleep disorders were not investigated.

As part of an epidemiologic study of psychiatric disorders carried out in 1995, several sleep complaints were investigated in a representative general community sample in Mexico City. In this paper, we present the prevalence estimates of several complaints of sleep disturbances and the corresponding rates accounting for the coexistence with other sleep-related symptoms.

## MATERIALS AND METHODS

### Sample

Data for this study was derived from the Epidemiology of Psychiatric Comorbidity Project (EPM) carried out in 1995. The design of the study was a household survey restricted to Mexico City, excluding the rest of the metropolitan area. The target population was adults aged 18 to 64 years living permanently or temporarily in private dwellings in one of the 16 administrative divisions of the city. The sampling design was multistage and stratified by sex and the availability of mental health services in the administrative divisions of the city. Two domains were defined based on the existence of mental health services: eight administrative divisions with them and eight without them.

The primary sampling unit corresponded to the geostadistic basic area (AGEB) defined for the XI General Population and Household Census in 1990. Independently, in each domain 48 AGEBs were selected with proportional probability relative to the size defined as the number of dwellings in each AGEB in accordance to the 1990 census. Finally, 96 urban AGEBs were selected.

At the second sampling stage, for each selected AGEB, a map showing numbered blocks of houses bounded on every side by a street was obtained; these represented the second sampling unit within each selected AGEB. Six blocks were selected with equal probability from each AGEB, obtaining a total of 288 for each domain.

A detailed sketch of each selected block was done, clearly identifying private dwellings. These were grouped in segments of approximately seven dwellings as a mean. These segments represented the third sampling unit. Systematic sampling was carried out in order to select a total of 576 segments in each domain. The selection probability in the third stage resulted in a self-weighted segment sample within each domain. Also, during the administration of the home questionnaires, a census of all households within each selected segment was carried out; thus, the selection probability of each household was equal to the segment obtaining a self-weighted sample within each domain.

The last sampling stage selected one subject in each dwelling, looking for equal numbers of females and males within the selected dwellings. Values for factor expansion were obtained at the end of the field work.

During the first phase, 4603 households were visited, obtaining a response rate of 71.7% (n=3300); the non-response was associated with informant absence, refusal to give information, abandoned household or the place was not a household. On the second phase, where individual interviews were carried out, out from 3200 eligible 18 to 65 year-old subjects, complete interviews were obtained from 1933; all participants provided informed consent. The response rate was 60.4%. Non-response associated with the informant represented 39.4%, mainly because they were reported as temporary absent or nobody was at home after repeated visits (at least four) in different hours and days. Only 8% openly refused the interview. The self-weighted sample by gender and age group was not significantly different from 1990 population census. The project was approved by the Institutional Research Committee. Details on the study design have been published elsewhere^14^.

### Instrument

The battery administered in the EPM consisted of thirteen sections. The first section contained 14 questions on sleep disturbances. These questions were taken from a non-validated Spanish version of the Sleep Disorders Questionnaire (SDQ)^15^, an instrument developed to distinguish patients with a high risk of having a sleep disorder. Selected SDQ items were those pertaining to symptoms of snoring, insomnia, obstructive sleep apnea, restless legs syndrome, sleep paralysis, diurnal excessive sleepiness, narcolepsy and hypnotic use. The response options for each question in the SDQ follow a frequency scale (never, seldom, sometimes, often, and always). The whole survey was carried out through face-to face interviews conducted by trained interviewers.

On the basis of the responses to the selected items, several sleep disturbances were defined. Snoring was considered positive when it occurred "often" or "always" and/or when snoring that bothers others occurred "often" or "always". "Sometimes" to "always" answers to "Do you feel sleepy during the day?" were considered indicative of complaints of excessive daytime sleepiness (EDS).

Obstructive sleep apnea syndrome (OSAS) complaints consisted of the combination of snoring ("sometimes" to "always" answers for snoring that bothers others and/or "often" or "always" for snoring) plus EDS. Complaints of restless legs syndrome (RLS) were considered present when a sensation of restless legs while falling asleep occurred "often" or "always". Sleep paralysis was defined as feeling "seldom" to "always" paralyzed upon awakening.

Complaints of narcolepsy were defined as feeling "often" or "always" sleepy during the day plus a response of "seldom" to "always" for the experience of sudden muscular weakness when laughing. Insomnia complaints consisted of: a) difficulty initiating sleep (DIS) when trouble to fall asleep was experienced "sometimes" to "always" and, b) difficulty maintaining sleep (DMS) when waking up during the night was experienced "sometimes" to "always". Use of hypnotics was considered positive if drug consumption as an aid to sleep occurred "sometimes" to "always".

### Statistical Analysis

The internal consistency of the scale was measured using Cronbach's alpha coefficient. Further, cut-off points to define "caseness" were established at the 80^th^ and 90^th^ percentiles of the score distribution from the scale on the population. Likewise, in order to establish the face validity and potential clinical significance of the cut-off points, association with known significant psychosocial and medical variables (subjective health evaluation, couple's relationship, number of work hours per week, tension at work, score on the 12-item General Health Questionnaire ≥ 5, and any current chronic medical disease) related to sleep disorders was explored using a logistic regression analysis.

Analyses were done using the Stata 13.0 program. Prevalence and confidence interval estimation for the whole sample as well as for sex and age sub-populations accounted for the complex stratified sampling of the survey. Differences among prevalence of sleep complaints were considered statistically significant if their CI did not interact. Estimates of prevalence for each sleep disorder adjusted for a score at the 80^th^. percentile or higher in the questionnaire were also calculated.

## RESULTS

Table 1 presents the sociodemographic characteristics of the whole sample. The 14-item questionnaire showed a satisfactory reliability coefficient (Cronbach's alpha=0.78). Association between the cut-off points for the 80^th^ percentile (≥19) and for the 90^th^ percentile (≥21) and selected related variables is shown on Table 2. Prevalence estimates for the different sleep disturbances categories and adjusted for the coexistence of sleep-related symptoms are shown in Tables 3 and 4, respectively.

**Table 1:** Demographic characteristics of the sample.

	Female (n=1062)	Male (n=871)	Total (n=1933)
Age (years)	35.2 (.46)	34.2 (.50)	34.8 (.38)
Mean (SE)			
Education (years)	9.0 (.12)	10.1 (.22)	9.5 (.14)
Mean (SE)			
Marital status (%)			
Single	22.9	26.7	24.6
Married/With partner	60.3	67.6	63.6
Divorced/Separated	10.5	4.5	7.7
Widowed	6.1	1.0	3.8

**Table 2:** Association between sleep problems and selected related variables.

PERCENTIL	8^TH^ DECIL > 19 OR (IC 95%)	9^TH^ DECIL > 21 OR (IC 95%)
Health status	1.2 (1.01-1.33)	1.2 (1.1-1.4)
Couple's relationship	1.1 (0.94-1.4)	1.3 (1.04-1.6)
Work hours per week	1.0 (0.99-1.0)	1.0 (0.99-1.0)
Tension at work	1.7 (1.2-2.5)	1.6 (1.3-2.1)
GHQ > 5	1.4 (1.3-1.5)	1.4 (1.3-1.5)
Chronic medical illness	2.5 (1.9-3.2)	2.5 (1.8-3.4)

GHQ=General Health Questionnaire

**Table 3:** Prevalence of complaints of sleep disturbances by age group and gender.

	Age Group					Total (n=1933)
	18-24 years (n=364) % (95% CI)	25-34 years (n=632) % (95% CI)	35-44 years (n=478) % (95% CI)	45-54 years (n=260) % (95% CI)	55-65 years (n=199) % (95% CI)	% (95% CI)
Snoring						
Male	6.1 (2.1-16.4)	11.7^†^ (8.2-16.4)	17.5^†^ (12.9-23.4)	13.4 (6.7-24.7)	39.9 (25.9-55.6)	14.2^†^ (10.5-18.8)
Female	1.5 (0.6-3.7)	3.0 (1.6-5.4)	5.0 (2.5-9.7)	18.0 (10.7-28.5)	19.3 (10.1-33.7)	6.7 (4.8-9.4)
Total	3.7 (1.6-8.5)	6.8 (4.8-9.7)	9.7 (7.2-12.9)	16.1 (11.5-22.1)	28.4 (18.5-40.8)	9.9 (8.2-12.1)
EDS						
Male	13.4 (8.9-17.8)	20.6 (14.9-27.7)	12.0 (8.1-17.5)	17.5 (9.8-29.3)	31.7 (19.4-47.4)	17.8 (14.1-22.2)
Female	13.2 (9.0-19.0)	21.7 (17.0-27.3)	27.1 ^†^ (20.7-34.7)	33.6 ^†^ (25.1-45.4)	28.5 (20.5-38.1)	23.3 (21.2-25.5)
Total	13.3 (10.1-17.4)	21.2 (17.5-25.3)	21.5 (17.2-26.6)	27.1 (19.6-36.2)	29.9 (22.8-38.1)	20.9 (19.0-22.9)
OSAS						
Male	2.3 (0.8-6.6)	8.4 (4.8-14.2)	6.8 (3.5-12.6)	16.0 (6.7-33.7)	34.9 (17.9-56.8)	9.2 (5.9-14.0)
Female	1.2 (0.5-2.8)	3.4 (1.6-7.1)	6.2 (3.4-10.9)	17.8 (10.8-27.9)	19.4 (11.3-31.4)	6.7 (5.2-8.6)
Total	1.7 (0.8-3.6)	5.4 (3.3-8.8)	6.4 (3.7-10.8)	17.2 (11.2-25.4)	25.4 (15.7-38.3)	7.7 (5.9-10.0)
RLS						
Male	2.6 (0.7-8.8)	3.2 (2.0-5.1)	2.5 (1.0-5.7)	0.4 (0.06-3.5)	4.1 (0.5-25.3)	2.7 (1.7-4.2)
Female	4.0 (1.5-10.4)	2.1 (1.2-3.7)	(4.0-11.4)	9.5 ^†^ (5.0-17.3)	13.4 (8.4-20.6)	5.7 ^†^ (4.4-7.4)
Total	3.3 (1.3-8.0)	2.6 (2.0-3.4)	5.2 (3.4-7.9)	5.8 (3.0-11.0)	9.3 (5.4-15.5)	4.4 (3.4-5.7)
Narcolepsy						
Male	0	0	0	1.1 (0.2-5.3)	0	0.1 (0.02-0.7)
Female	0	1.4 (0.4-4.4)	1.6 (0.5-4.2)	2.3 (0.6-8.5)	5.0 (1.3-17.5)	1.6 ^†^ (1.0-2.5)
Total	0	0.8 (0.2-2.4)	0.9 (0.3-2.6)	1.8 (0.6-5.3)	2.8 (0.7-9.9)	0.9 (0.6-1.5)
Insomnia						
Male	38.4 (32.7-44.6)	33.5 (26.2-41.6)	28.3 (22.4-35.0)	45.5 (34.9-56.5)	46.8 (32.6-61.4)	36.7 (32.9-40.7)
Female	33.6 (36.9-41.1)	39.0 (33.0-45.4)	39.0 (31.7-46.9)	60.8 (48.2-71.1)	51.2 (37.9-64.4)	41.9 (38.4-45.5)
Total	35.9 (31.4-40.7)	36.5 (31.3-42.1)	35.0 (29.8-40.7)	54.6 (44.4-64.5)	49.2 (40.5-58.1)	39.7 (36.9-42.5)
DIS						
Male	23.4 (16.4-32.3)	22.9 (17.1-29.8)	12.7 (8.4-18.8)	32.3 (22.8-43.5)	22.8 (14.3-34.4)	22.4 (19.2-25.9)
Female	20.1 (13.4-29.1)	23.7 (17.7-31.0)	28.5 ^†^ (20.3-38.4)	40.6 (29.5-52.9)	38.5 (26.4-52.2)	27.7 (23.7-32.1)
Total	21.7 (16.6-27.8)	23.3 (18.4-29.0)	22.6 (17.2-29.2)	37.3 (29.2-46.1)	31.6 (24.2-40.0)	25.4 (22.5-28.5)
DMS						
Male	28.3 (22.2-35.3)	21.7 (15.1-30.3)	23.9 (18.4-30.4)	32.2 (23.5-42.2)	38.5 (24.3-55.0)	26.9 (23.8-30.2)
Female	23.4 (18.1-29.8)	32.1 (25.5-39.4)	31.9 (24.9-39.9)	53.0 (41.6-64.1)	44.9 (31.8-58.8)	34.1 ^†^ (30.7-37.7)
Total	25.7 (22.1-29.8)	27.5 (22.9-32.6)	28.9 (23.6-35.0)	44.5 (35.4-54.1)	42.1 (32.2-52.6)	31.0 (28.6-33.6)
Hypnotics						
Males	0	0.5 (0.1-2.2)	2.8 (1.0-7.7)	0.2 (0.03-2.0)	2.5 (0.7-7.6)	0.9 (0.4-2.1)
Females	0	0.6 (0.1-2.2)	3.9 (1.9-7.8)	3.7 (1.3-10.1)	1.4 (0.4-4.4)	1.7 (1.0-2.9)
Total	0	0.6 (0.2-1.6)	3.5 (1.8-6.7)	2.3 (0.8-6.2)	1.9 (0.7-4.7)	1.4 (0.9-2.2)
SP						
Male	19.2 (13.1-27.2)	9.5 (7.0-12.6)	6.7 (4.5-10.0)	10.5 (5.7-18.3)	18.0 (9.7-30.9)	12.6 (10.1-15.8)
Female	13.2 (9.1-18.9)	7.7 (5.3-11.1)	17.3^†^ (12.8-23.1)	16.0 (8.6-27.9)	20.5 (11.9-32.9)	13.6 (11.5-16.0)
Total	16.1 (12.8-20.0)	8.5 (6.6-10.8)	13.4 (10.2-17.4)	13.8 (8.3-22.0)	19.4 (14.0-26.1)	13.2 (11.5-15.1)

† p< .01 with the same age group; OSAS=obstructive sleep apnea syndrome; RLS=restless legs syndrome; SP=sleep paralysis; EDS=excessive diurnal sleepiness; DIS=difficulty initiating sleep; DMS=difficulty maintaining sleep.

**Table 4:** Prevalence of complaints of sleep disturbances by age group and gender adjusted for percentile 80th.

	Age Group					Total (n=1933)
	18-24 years (n=364) % (95% CI)	25-34 years (n=632) % (95% CI)	35-44 years (n=478) % (95% CI)	45-54 years (n=260) % (95% CI)	55-65 years (n=199) % (95% CI)	% (95% CI)
Snoring						
Male	3.3 (0.6-15.8)	7.9 (4.6-13.1)	10.0 (6.9-14.2)	10.3 (4.5-21.8)	28.0 (14.9-46.2)	9.3 (5.8-14.5)
Female	1.5 (0.6-3.7)	2.8 (1.4-5.4)	4.0 (1.9-8.2)	16.6 (9.7-27.0)	16.6 (8.9-28.6)	6.0 (4.3-8.3)
Total	2.3 (.7-7.4)	5.1 (3.3-7.7)	6.3 (4.3-9.1)	14.0 (9.4-20.4)	21.6 (13.3-33.1)	7.4 (5.6-9.8)
EDS						
Male	3.2 (1.2-8.0)	8.9 (5.9-13.2)	6.3 (3.7-10.6)	11.7 (4.9-25.2)	19.8 (9.5-36.7)	8.3 (5.9-11.5)
Female	6.0 (3.3-10.7)	7.1 (4.4-11.3)	15.0 (10.3-21.5)	20.2 (12.6-30.6)	22.2 (15.2-31.2)	11.9 (9.8-14.2)
Total	4.7 (2.7-7.9)	7.9 (5.7-10.8)	11.8 (8.6-16.0)	16.7 (10.6-25.3)	21.1 (14.7-29.3)	10.3 (8.6-12.3)
OSAS						
Male	0.3 (0.04-2.9)	4.7 (2.8-7.8)	3.6 (1.6-7.8)	9.2 (2.9-25.1)	22.0 (10.1-41.7)	5.3 (3.1-8.9)
Female	0.8 (0.3-2.0)	1.3 (0.4-4.0)	5.1 (2.8-9.1)	13.6 (7.6-23.3)	14.6 (7.7-25.9)	4.9 (3.5-6.8)
Total	0.6 (0.2-1.4)	2.8 (1.6-4.9)	4.6 (2.6-7.8)	11.8 (6.8-19.7)	17.7 (10.7-27.8)	5.0 (3.5-7.1)
RLS						
Male	2.6 (0.7-8.8)	3.2 (2.0-5.1)	2.3 (0.9-5.4)	0.4 (0.06-3.5)	4.1 (0.5-25.3)	2.6 (1.7-4.1)
Female	3.4 (1.1-10.4)	0.9 (0.3-2.7)	5.7 (3.6-8.8)	7.7 ^†^ (3.8-14.9)	7.7 ^†^ (3.8-14.9)	4.6 (3.4-6.2)
Total	3.0 (1.1-7.8)	1.9 (1.4-2.7)	4.4 (3.0-6.5)	4.7 (2.3-9.5)	8.8 (5.0-15.2)	3.8 (2.8-5.0)
Narcolepsy						
Male	0	0	0	1.1 (0.2-5.3)	0	0.1 (0.02-0.7)
Female	0	1.4 (0.4-4.4)	1.1 (0.3-3.5)	2.3 (0.6-8.4)	5.0 (1.3-17.5)	1.5 ^†^ (0.8-2.6)
Total	0	0.8 (0.2-2.4)	0.7 (0.2-2.1)	1.8 (0.6-5.3)	2.8 (0.7-9.9)	0.9 (0.5-1.5)
Insomnia						
Male	10.0 (5.6-17.4)	14.3 (9.9-20.3)	9.4 (6.0-14.2)	23.4 (16.3-32.4)	25.4 (14.2-41.1)	14.4 (11.5-18.0)
Female	10.3 (5.7-17.9)	12.7 (8.9-17.8)	23.0 ^†^ (17.1-30.3)	34.1 (25.1-44.4)	33.4 (25.9-41.8)	19.5 (16.2-23.2)
Total	10.2 (6.5-15.4)	13.4 (10.3-17.3)	17.9 (13.7-23.2)	29.8 (23.2-37.3)	29.9 (23.4-37.2)	17.3 (14.8-20.1)
DIS						
Male	7.7 (3.6-15.4)	11.1 (7.4-16.2)	5.5 (2.7-11.0)	20.8 (14.1-29.7)	17.8 (9.1-31.8)	11.0 (8.8-13.6)
Female	9.5 (4.8-18.1)	11.3 (7.2-17.2)	22.1 ^†^ (14.8-31.5)	31.1 (19.9-45.0)	31.0 (21.5-42.4)	18.1 ^†^ (14.6-22.1)
Total	8.7 (5.1-14.3)	11.2 (8.2-15.0)	15.9 (11.0-22.4)	27 (19.4-36.1)	25.0 (18.3-33.2)	15.0 (12.6-17.7)
DMS						
Male	10.8 (5.8-19.3)	13.0 (7.9-20.7)	10.3 (6.7-15.4)	24.1 (16.6-33.7)	25.7 (13.4-43.5)	14.6 (11.7-18.0)
Female	8.2 (3.9-16.5)	13.9 (9.3-20.3)	22.2 ^†^ (15.8-30.2)	39.8 (29.4-51.3)	34.5 (24.9-45.6)	20.0 (16.7-23.9)
Total	9.4 (5.9-14.7)	13.5 (9.5-18.7)	17.9 (13.3-23.5)	32.9 (25.4-41.4)	30.7 (22.1-40.9)	17.6 (15.1-20.5)
Hypnotics						
Males	0	0.5 (0.1-2.2)	2.8 (1.0-7.7)	0.2 (0.03-2.0)	2.5 (0.7-7.6)	0.9 (0.4-2.1)
Females	0	0.6 (0.1-2.2)	3.9 (1.9-7.8)	3.7 (1.3-10.1)	1.4 (0.4-4.4)	1.7 (1.0-2.9)
Total	0	0.6 (0.2-1.6)	3.5 (1.8-6.7)	2.3 (0.8-6.2)	1.9 (0.7-4.7)	1.4 (0.9-2.2)
SP						
Male	8.2 (4.3-15.2)	6.4 (4.0-9.9)	4.9 (2.8-8.5)	5.8 (2.5-12.9)	15.1 (7.4-28.5)	7.4 (5.3-10.3)
Female	8.7 (4.6-15.9)	4.6 (2.8-7.5)	11.7 (7.6-17.4)	13.3 (6.7-24.7)	18.0 (10.6-28.9)	9.7 (7.4-12.5)
Total	8.5 (5.4-13.2)	5.4 (3.8-7.7)	9.2 (6.5-12.7)	10.2 (5.5-18.2)	16.7 (12.0-22.9)	8.7 (7.2-10.6)

† p< .01 with the same age group; OSAS=obstructive sleep apnea syndrome; RLS=restless legs syndrome; SP=sleep paralysis; EDS=excessive diurnal sleepiness; DIS=difficulty initiating sleep; DMS=difficulty maintaining sleep.

Snoring was reported by 9.9% of the sample; the prevalence was significantly higher in men than in women and increased with age. The prevalence of EDS complaints was 20.9%; in contrast to snoring, EDS was more frequent among women but it also showed an increasing pattern with age.

Complaints of obstructive sleep apnea were found in 7.7% of the sample; interestingly, there were no significant gender differences.

The prevalence of RLS complaints was 4.4%. This rate was significantly higher in women than in men and increased with age. The gender difference was particularly higher in the middle-aged group. The prevalence of narcolepsy with cataplexy complaints (NC) was 0.9%; again, NC was significantly more frequent among women (1.6%) than in men (0.1%) and higher in the oldest women.

Insomnia complaints were reported by 39.7% of the sample. The prevalence was higher in women (41.9%) than in men (36.7%) and increased with age but the difference was not significant. Difficulty initiating sleep (DIS) and difficulty maintaining sleep (DMS) were found in 25.4% and 31.0% of the sample, respectively. Insomnia complaints were higher in women (DIS 27.7%, DMS 34.1%) than in men (DIS 22.4%, DMS 26.9%), and they showed an increase with age. However, the difference was significant only for DMS rates. In spite of the high rates of insomnia, use of hypnotics was reported by only 1.2% of the sample. Finally, the prevalence for complaints of sleep paralysis was 13.2%, with a significant higher rate in 35-44 year-old women.

When the prevalence rates were calculated accounting for a score at the 80^th^ percentile or higher, the rates for all sleep disturbances categories decreased (Table 4). However, more notable reductions were observed for complaints of insomnia (17.3%), EDS (10.3%), and SP (8.7%), while minor decreases were observed for complaints of snoring (7.4%), OSAS (5%), and RLS (3.8%); NC prevalence practically did not change (0.9%). The gender differences in total rates for snoring, RLS, DMS lost significance while NC remained unchanged, and a significant higher prevalence of DIS in women emerged.

## DISCUSSION

This study shows, firstly, that complaints of sleep disturbances have a high frequency among adult residents in Mexico City; and, secondly, that more than a half of the individuals with a given sleep disturbance have symptoms in other sleep domains resulting in a global sleep disturbance associated to psychosocial and health impairments.

Insomnia complaints were the most common sleep disturbance. The prevalence of 39.7% is consistent with both the rates reported in other countries^16^ and the prevalence found in previous population-based studies in Mexican individuals (35% and 36.1%)^12,13^. As in most epidemiological studies, women were more likely to report insomnia symptoms and these increased with age. This gender difference was consistently significant in middle aged individuals. We also found that DMS was more frequent than DIS, a finding repetitively identified in other populations^17,18^.

Almost half of the subjects with insomnia complaints (17.3%) had also symptoms in other sleep domains; in other words, they had a more disturbed sleep. This means that about one out of five Mexican adults requires a sleep-focused clinical assessment. This percentage of insomnia subjects with a more disturbed sleep is singularly similar to the prevalence of insomnia symptoms accompanied by daytime consequences; therefore, the contribution of other sleep-related symptoms to the daytime consequences of insomnia should be addressed in future studies.

Interestingly, in spite of the high frequency of insomnia, the use of hypnotic drugs was low. Previous studies conducted in Mexico^12,13^ and in other countries have reported prevalence rates between 0.7% and 8% for the current use of hypnotics, irrespective of the frequency of consumption^19-21^. However, in one of the Mexican studies^12^, when only individuals who frequently used hypnotics were considered, the prevalence dropped to 1.4%, a figure identical to the one we have found. The prevalence rates of hypnotic consumption did not change when global sleep symptoms were taken into account; this may imply that individuals who use hypnotic medications have a more disturbed sleep.

Sedative use rates vary among different countries. The prevalence in Mexico is similar to the one reported in the United Kingdom^19^, higher than in both Germany and Italy^19^, but lower than in other Latin American countries^13,20^, France^19^, Norway^21^, Canada^22^, and the United States^23^. The consistent high rates of insomnia symptoms, the relatively low prevalence of hypnotics use, and the recently found risks associated to these drugs^24,25^ indicate that we might be in a timely situation to spread the use of behavioral therapies with well-proven safety and efficacy^26,27^, such as stimulus control therapy, sleep restriction therapy, and multicomponent treatments.

Complaints of EDS were the second most frequent sleep disturbance in the whole sample. The prevalence we report is similar to the rate of 21.5% found by Téllez López et al.^12^ who also investigated EDS in terms of frequency. Likewise, the prevalence we report was slightly higher than the 17.7% reported by Torre-Bouscoulet et al.^13^ although these authors defined EDS as its presence during all or most of the days. As it was the case with insomnia complaints, we found that the EDS prevalence rate dropped to a half when the presence of additional sleep-related symptoms was considered; however the rate remains high. According to our results, the sources of EDS might be different between females and males, particularly in the 35- to 54-year old group, because women were significantly more affected by insomnia, RLS, and use of hypnotics, while men showed higher rates of complaints of snoring and OSAS.

Depending on the measurement method and the characteristics of the surveyed sample, snoring prevalence rates range from as low as 7% to as high as 85%^28,29^. The estimates we obtained are near the lowest end, probably due to the inclusion of young individuals in the sample. Nevertheless, well-known factors associated to snoring, such as male gender and age, were replicated in the crude prevalence estimates. Interestingly, when rates were adjusted for the presence of symptoms in other sleep domains, the gender difference lost significance.

In relation to this, two findings of snoring in women deserve a special mention: 1) the dramatic increase in the prevalence rates observed in 45-54 year old women in comparison with the younger groups; and 2) the scarce reduction in the adjusted prevalence rates. Taken together, these results suggest that the emergence of snoring in women may have a more pathologic meaning. In fact, in the Sleep Heart Health Study, Young et al.^30^ found that women with habitual snoring (3-7 nights/week) had a higher odds ratio for an AHI of 15 than men with habitual snoring.

Regarding to the prevalence estimates of OSAS complaints, we must underline that these were based on only two symptoms (EDS and habitual snoring) when the gold standard for diagnosis is the polysomnography. However, a study in Mexican population has documented that the combination of snoring and EDS has a low sensitivity and positive predictive value (PPV), but a high specificity and negative predictive value (NPV), considering a Respiratory disturbance Index ≥ 15 events/h^13^. In this way, based on the presence of these two classical symptoms of OSA, we found crude and adjusted prevalence rates of 7.7 and 5%, respectively. These figures are in the estimated prevalence range of 6 to 20% for mild or moderate OSAS in other countries^31^.

However, they are higher than the prevalence estimates of 3.2% for the combination of snoring/sleepiness/observed apneas and of 4.9% for snoring/observed apneas, previously found in an adult sample of Mexico City^13^. The difference may be due to the inclusion of witnessed apneas in the OSAS definition used by the authors which may have resulted in a higher specificity. High prevalence rates found in recent studies^32,33^ have called into question current definitions of sleep apnea syndrome.

In the HypnoLaus study^32^, conducted in a large population-based sample of adults of Lausanne, Switzerland who underwent full polysomnography, an apnea/hypopnea index (AHI) ≥ 5 events per h (according to American Academy Sleep Medicine 2013 scoring criteria^34^) was observed in 83.8% and 60.8% of men and women, respectively; whereas an AHI ≥ 15 was found in 49.7% of men and 23.4 % of women. In the Sao Paulo Epidemiologic Study^33^, the prevalence for OSAS (using the ICSD-2 criteria) was also high, 46.6% in men and 46.6% in women. Furthermore, when the authors of the HypnoLaus study calculated the prevalence of OSAS according to the International Classification of Sleep Disorders (ICSD-3) criteria they obtained an "unrealistically" high rate: 74.7% in men and 52% in women^35^.

Heinzer et al. have pointed out that ICSD-3 OSAS definition might be too inclusive. They suggest that symptoms and comorbidities should only be taken into account when they are clearly related to OSAS. Indeed they found a prevalence of 12.5% in men and 5.9% in women when OSAS was defined as the presence of excessive daytime sleepiness and an AHI of 5 events per h or more. In this sense, in the present study we observed a drop of the prevalence, specifically in the male group, when the prevalence rate was adjusted for the coexistence of disturbed sleep associated with psychosocial/health impairments. This might be congruent with the proposal of Heinzer et al. of considering OSAS as a disease with a severity spectrum in terms of the risk for developing comorbidities.

In contrast to most of the literature that indicates OSAS is more frequent in men with a gender ratio of 2:1, we found no significant gender differences in overall and age group prevalence. This is a provocative and hard to explain finding. In one of the most rigorous epidemiological studies, Durán et al.^36^ using a parameter of an AHI ≥ 5 for the presence of OSAS, reported a comparable prevalence between men and women (26.2% and 28%, respectively); however, higher rates for men were present in the age group analysis as in many other studies.

In consequence, our findings might suggest that Mexican population shows a different profile where the predominance of men is not so strong. In support of this view are the results of Bouscoulet et al.^13^ who studied a subsample of Mexico City adult residents with a simplified respiratory polygraphy and found that male gender was not a significant risk factor for OSAS defined as an Epworth Sleepiness Scale score ≥ 11 and a Respiratory Disturbance Index ≥ 15 events/h. Research with stricter methodology in Mexican population is needed to elucidate this intriguing issue. Unlike the gender related negative results, we observed an increase of prevalence with age, in such a way that one out of four adults between 55 and 65 years old would need to be assessed with polygraphic monitoring during sleep because of the high risk of OSAS.

As far as we know, this is the first study providing an estimate of prevalence of RLS complaints in a representative sample of Mexican individuals. A population-based study conducted in a sample of Mexican female teachers reported that 15.6% met RLS diagnostic criteria and 8.3% presented with RLS symptoms at least once per week^37^. Both prevalence rates are higher than those we found in the female subsample of our study.

When we compare our results with the prevalence estimates reported in studies conducted in general population^38^, the rate we obtained is near the lowest limit of the prevalence range, even if we only take into account the studies which similarly used a single question to assess RLS. Low prevalence rates have also been reported in Asian countries. However, in contrast with the lack of age-related change in rates found in those studies, we observed that the prevalence of RLS complaints in women increased steadily with age, while in men the prevalence appeared to remain mostly unchanged. Yet, we set the frequency of RLS complaints at "often" or "always", and this parameter has been found to influence prevalence in such a way that the presence of RLS symptoms once or twice per week decreases the prevalence by about 40%-50%^38^.

The prevalence of narcolepsy complaints we report is high in comparison with other studies. In a recent review about the epidemiology of narcolepsy, it was pointed out that estimates of prevalence are high when the identification of cases is based solely on a symptom screen without more in-depth assessment^39^. As this was the case in our study, the rate might be inflated by false positives; thus, the higher prevalence of narcolepsy found in women may be due to the gender difference in the rates of EDS. Future studies in our population should include follow-up intensive screening of initial positive cases.

Few studies have examined SP rates in population-based samples. Ohayon et al.^40^ reported a prevalence of 6.2% in a representative sample of general population (age ≥ 15 years) from Germany and Italy, while Téllez López et al.^12^ found a SP rate of 12.5%. The prevalence we have found is very similar to the one reported by Téllez López et al.^12^ in Mexican subjects from a different city in our country. Interestingly, rates of SP complaints were higher in the youngest and the oldest age groups.

Young individuals have repetitively been found as one of the groups more affected by SP^41^, but reports of SP in elderly people are scarce. Wing et al.^42^ found a high frequency of SP in a sample of Chinese subjects and Ohayon et al.^40^ reported that SP could occur at any age. Considering that SP has been associated to fragmented sleep, an association to aging does not seem to be surprising. We observed a smaller reduction of the prevalence rate in the oldest group when it was adjusted for the coexistence of sleep symptoms in other domains, which could be suggesting that SP may be associated to a more disturbed sleep; however, the role of SP or its influence over global sleep quality of old individuals is unknown.

Definitely, this topic warrants future research. On the other hand, frequency of SP episodes is of particular relevance because it might be related to a higher impact on both sleep and life quality. In this sense, we have found that only 1.4% (female 1.9% vs. male 0.7%; χ^2^=4.6, *p*=.18) of the whole sample experienced frequent SP events. This low figure might well correspond to the "true" SP disorder, which is infrequently seen in clinical settings.

Some limitations of this study must be recognized. Firstly, as the principal objective of the study was to estimate the 12-month and lifetime prevalence of common psychiatric disorders in the adult population, only persons aged 18 to 64 years were included. Since elderly people have high prevalence of sleep symptoms, the prevalence for several sleep disorders may be underestimated. Secondly, although a relatively low response rate of 60.4% was obtained, it does not invalidate results presented for the whole population as well as for sex and age-groups in accordance to the population census of 1990.

Considering a fortuitous sampling without replacement the highest sample size was 384 subjects. However, as a multistage cluster design was employed, a correction factor of 2 was estimated based on other health surveys, so that the corrected sample size representative of the whole population would have been 768 subjects. Further, as an equal number of women and men were defined and availability of mental health services was not a significant domain for the purpose of this paper, a sample size of 1536 subjects was enough to obtain the estimated precision for the total prevalence in regards of sleep disturbances.

In addition, some of our data are consistent with previous population-based studies in Mexican and other populations. Thirdly, the survey was carried out in 1995 and during the last two decades some risk factors associated to sleep-related symptoms have changed; therefore some prevalence rates might have also changed.

For example, overweight and obesity - consistent risk factors associated with OSAS - have a very high prevalence in Mexican population (38.8% and 32.4%, respectively)^43^. The prevalence rate for a body mass index ≥ 25 kg/m^(2 )^is slightly higher in women (73.0%) compared to men (69.4%) and 40-59 year old adults are the most affected age group. Moreover, during the 2000 - 2012 period, both overweight and obesity have shown a pattern of increasing prevalence rates. In this way, given the positive relation between BMI and OSAS, current prevalence of OSAS in Mexico may be higher. Fourthly, we used only one or a few questions to define sleep disturbances which might not have the sensitivity to capture the complexity of sleep disorders. In spite of these limitations, we believe this study provides relevant information about sleep disturbances in Mexican population.

## CONCLUSIONS

In general, our results show that at least one third of the Mexicans suffers from sleep-related symptoms. In the light of this, the implementation of strategies directed to preventing sleep disturbances and improving sleep are needed. These should include education and promotion of healthy sleep habits among the population, appropriate health infrastructure with human and material resources (sleep clinics), and opportune access to health services.
